# Renal tissue pro-inflammatory gene expression is reduced by erythropoietin in rats subjected to hemorrhagic shock

**DOI:** 10.15171/jnp.2017.12

**Published:** 2016-11-29

**Authors:** Mina Ranjbaran, Mehri Kadkhodaee, Behjat Seifi

**Affiliations:** Department of Physiology, Faculty of Medicine, Tehran University of Medical Sciences, Tehran, Iran

**Keywords:** Hemorrhagic shock, Kidney, Erythropoietin, Gene expression, Cytokines

## Abstract

**Background:**

Hemorrhagic shock (HS) is a condition produced by considerable loss of intravascular volume, which may eventually lead to organ damage and death.

**Objectives:**

In the present study, the potential implication of the kidney tissue tumor necrosis factor-α (TNF-α), interleukin-6 (IL-6), and interleukin-10 (IL-10) were evaluated in the protective effects of erythropoietin (EPO) during HS.

**Materials and Methods:**

Male Wistar rats were randomized into three experimental groups; Sham, HS (hemorrhagic shock and resuscitation), and EPO (erythropoietin). HS was induced by 50% blood volume hemorrhage over 30 minutes. After 2 hours, resuscitation was performed within 30 minutes. In the EPO group, EPO (300 IU/kg, i.v.) was administered 10 minutes before HS induction. Urine was collected to determine urinary N-acetyl-β-D-glucosaminidase (NAG) activity level. The kidney cytokines (TNF-α, IL-6 and IL-10) mRNA expressions were measured by real-time polymerase chain reaction (PCR).

**Results:**

HS rats showed significant increase in urinary NAG activity compared to the sham group. EPO significantly attenuated the rises in urinary NAG activity compared to the HS group. In the HS animals, renal TNF-α and IL-6 mRNA expressions increased whereas no difference was observed in IL-10 mRNA expression between the HS and sham groups. EPO was able to decrease renal TNF-α and IL-6 production and increase IL-10 mRNA expression.

**Conclusions:**

In this study, we demonstrated that EPO attenuates kidney damage in rats subjected to HS. The beneficial effects of EPO may be at least partly mediated by modifications in the inflammatory cascade.

**Implication for health policy/practice/research/medical education:**

In this experimental model, we found that erythropoietin (EPO) is able to decrease the renal injury induced by hemorrhagic shock (HS). EPO exerts its beneficial effects on the kidney tissues, in part, due to modifications in the inflammatory cascade.

## 1. Background

Hemorrhagic shock (HS) is one of the main causes of morbidity and mortality among trauma patients (1). During HS, hemodynamic instability, reduced oxygen delivery and low tissue perfusion may eventually cause cellular hypoxia, multiple organ failure and death (1). In many cases, renal dysfunction occurs following HS because the kidney function directly depends on the renal perfusion pressure. The resultant hypoxia exacerbates renal injury which eventually leads to acute kidney injury (2).

One of the major complications of HS is related to the abnormal and deleterious activation of the immune system, which may manifest as a relatively pro-inflammatory state (1). After HS, nuclear factor-κB (NF-κB) activates the inflammatory cascade. Exacerbating production of pro-inflammatory cytokines like tumor necrosis factor-α (TNF-α) or interleukin-6 (IL-6) may result to the severe injury to the body organs (3,4).

EPO was originally described as a glycoprotein hormone required for erythropoiesis. However, recent studies indicated that the therapeutic benefits of EPO are beyond anemia correction (5). EPO has tissue-protective effects in many organs including the kidney (5). Several studies have shown that EPO exerts anti-apoptotic, anti-oxidant and angiogenic effects in animal models of ischemia-reperfusion injury (6-8).

## 2. Objectives

Discovering of the effects of EPO on the inflammatory pathway is interesting. The major object of this study was to test the hypothesis that EPO is able to suppress immune system and prevent kidney damage against HS. To achieve this object, we evaluated pro- and anti-inflammatory cytokines mRNA expressions in the kidney tissues following HS induction and EPO administration in rats.

## 3. Materials and Methods

### 3.1. Animals

Animals were housed under standard conditions (12 hours light–dark cycle; 20–22°C) and were allowed food and water ad libitum.

### 3.2. Surgical procedure

Eighteen male Wistar rats (285–300 g) were randomly selected. Rats were anesthetized with ketamine (50 mg/kg) and xylazine (10 mg/kg) administered intraperitoneally. Left femoral artery and vein were cannulated by polyethylene catheters (PE-50). The arterial cannula was used for hemorrhage and the venous catheter was used for resuscitation and EPO administration.

### 3.3. HS protocol

The volume of hemorrhage was based on estimated 50% of total blood volume and calculated according to Ahmadi-Yazdi as follows: animal weight [g] × 0.03 + 0.7 mL (9).

To induce HS, 50% of the total blood volume was withdrawn using heparinized syringes over a period of 30 minutes through the arterial catheter. Two hours after the blood removal, resuscitation was performed with the shed blood and equal volume of Ringer’s lactate within 30 minutes via femoral vein. After resuscitation, animals were continuously monitored for a further 3 hours and scarified at the end of this time.

### 3.4. Experimental design

Animals were randomly allocated into 3 groups (n = 6): 1) Sham, anesthesia and surgery without induction of HS; 2) HS, hemorrhagic shock and resuscitation; and 3) EPO, rats were received 300 IU/kg recombinant human EPO (in 0.5 mL normal saline, i.v.) over 10 minutes before HS (4,10). At the end of the procedure, urine was collected from bladder to measure urinary N-acetyl-β-D-glucosaminidase (NAG) activity. Sections of the left kidneys were harvested for measurements of pro-inflammatory and anti- inflammatory cytokines by real-time polymerase chain reaction (PCR).

### 3.5. Renal functional assessment

Urinary NAG activity is a sensitive marker of early kidney tubular damage. The assay for urinary NAG activity is based on the enzymatic hydrolysis of p-nitrophenyl-N-β-D-acetyl-glucosaminide (11).

### 3.6. RNA extraction and Real-time PCR

Total RNA of kidney was isolated according to the manufacturer’s instructions (RNeasy Mini Kit; Qiagen). RNA concentration was determined using the NanoDrop^TM^ 1000 (Thermo Scientific, USA). Four micrograms of total RNA was reverse-transcribed into cDNA according to the manufacturer’s instructions (PrimeScript RT Master Mix, Takara, Japan).

Real-time PCR amplifications were conducted with the use of the ABI 7500 system (Applied Biosystems, USA). The reaction mixture contained 4 µL of diluted cDNA, 5 pm of each primer, 10 µL of 2X SYBR green master mixes in a total volume of 20 µL.

PCR was conducted at 95°C for 15 minutes, followed by 40 cycles at 95°C for 15 seconds, 58°C for 1 minute. This program was followed by analysis of melting curve that was performed with linear heating from 60-90°C.

This analysis was performed to measure TNF-α, IL-6 and IL-10 mRNA expressions in the kidney tissue samples. The amount of mRNA for each gene was normalized by the use of HPRT-1 (hypoxanthine phosphoribosyltransferase 1). PCR primers for all analyzed genes are shown in Table 1.

**Table 1:** Primers used for real-time PCR analysis

**Genes**	**Sense strand sequence**	**Anti-sense strand sequence**
TNF-α gene	GTGATCGGTCCCAACAAGGA	TGGTGGTTTGCTACGACGTG
IL-6 gene	AAGTCCGGAGAGGAGACTTCA	GCCATTGCACAACTCTTTTCTCATT
IL-10 gene	GACGCTGTCATCGATTTCTCC	AGTAGATGCCGGGTGGTTCA
HPRT-1 gene	CTCCTCAGACCGCTTTTCCC	AGCAAGTCTTTCAGTCCTGTCC

### 3.7. Ethical issues

The research followed the tenets of the Declaration of Helsinki. Experimental protocol and animal care methods in the experiments were approved by the Experimental Animal Committee of Tehran Medical Sciences University. Prior to the study, the protocol was confirmed to be in accordance with the Guidelines of Animal Ethics Committee of Tehran Medical Sciences University.

### 3.8. Statistical analysis

The data are presented as mean ± standard error of mean. One-way analysis of variance (ANOVA) was used to compare mean values between groups followed by Tukey’s post hoc test. *P* < 0.05 was considered statistically significant.

## 4. Results

### 4.1. Effects of EPO administration on urinary NAG activity during HS

**Figure 1:**
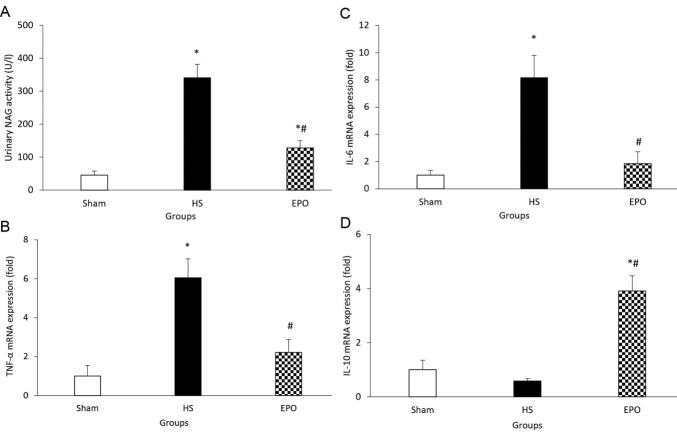
(A) Effect of EPO administration on urinary NAG activity during hemorrhagic shock. (B) Effect of EPO administration on renal TNF-α mRNA expression during hemorrhagic shock. Real-time PCR was used to assess the gene expression. (C) Effect of EPO administration on renal IL-6 mRNA expression during hemorrhagic shock. Real-time PCR was used to assess the gene expression. (D) Effect of EPO administration on renal IL-10 mRNA expression during hemorrhagic shock. Real-time PCR was used to assess the gene expression. Data are presented as the mean ± standard error of mean (n = 6). *P<0.05 compared to the sham group. # P< 0.05 compared to the HS group. Abbreviations: HS, hemorrhagic shock; EPO, erythropoietin+ HS; NAG, N-acetyl-β-D-glucosaminidase; TNF-α, tumor necrosis factor α; IL-6, interleukin 6; IL-10, interleukin 10.

HS significantly increased urinary NAG activity compared to the sham group (*P* < 0.05; Figure 1A). EPO administration significantly reduced this functional parameter compared to the HS group (*P* < 0.05; Figure 1A).

### 4.2. Effects of EPO administration on the pro-inflammatory cytokines mRNA expressions during HS

HS significantly increased renal TNF-α gene expression (*P* < 0.05; Figure 1B). In the EPO group, TNF-α mRNA expression was significantly reduced compared to the HS group (*P* < 0.05; Figure 1B).

In the HS group, IL-6 gene expression was significantly higher than those in the sham group (*P* < 0.05; Figure 1C). EPO administration significantly decreased IL-6 mRNA expression (*P* < 0.05; Figure 1C).

There was no significant difference in IL-10 mRNA expression in the HS group compared to the sham group (Figure 1D). In the EPO group, IL-10 gene expression was significantly increased compared to the sham and HS groups (*P* < 0.05; Figure 1D).

## 5. Discussion

HS is associated with a systemic inflammatory response and contributes to the pathophysiology of the multiple organ failure. In the present study, we evaluated the changes in the inflammatory cytokines using a fixed-volume model of HS following EPO administration.

Until recently, the key physiological role of EPO was thought to be the induction of erythropoiesis. However, a growing body of evidence shows that EPO has tissue-improving effects and reduces organ failure. EPO receptors have been found in vascular and non-vascular kidney tissues. Moreover, it seems that EPO is able to activate different pathways in the kidney tissue such as the Akt pathway, or heat shock protein (5).

In the present study, urinary NAG activity was measured in rats. In the HS group, NAG activity was significantly greater than the sham group. EPO significantly attenuated the rises in NAG activity compared to the HS group. NAG, as a lysosomal enzyme, is present in proximal tubular cells and determines renal injury in the early stages. Thus, urinary excretion of NAG is proportional to the degree of renal tubular cell breakdown (12). In our study, EPO treatment reduced the HS-induced rises in urinary NAG activity and improved renal tubular injury.

One of the major complications in HS is inflammatory cascade activation which leads to the development of organ damage. Acute phase of inflammation (innate immunity) is mediated through the activation of the immune system. During inflammation, mast cells and leukocytes are recruited to the site of injury (13). These inflammatory cells also produce mediators such as cytokines and chemokines, which intensify further recruiting inflammatory cells to the site of damage (14). These key mediators activate NF-κB, hypoxia-inducible factor-1α and various other factors which induce abnormal expression of inflammatory cytokines TNF-α and IL-6. In the present study, the potential involvement of renal cytokines was assessed following HS induction and EPO administration. In the HS group, mRNA expressions of TNF-α and IL-6 were significantly increased in the kidney tissue samples compared to the sham group. Renal TNF-α and IL-6 gene expressions were significantly decreased in EPO-treated animals. A pivotal intracellular pathway mediating the beneficial effects of EPO is the inhibition of pro-inflammatory cytokines. Similar results have been reported by Chen et al in 2007 (15). They showed that EPO reduces the expression of pro-inflammatory cytokines IL-1 and TNF-α in injured rat brain and is able to modify the cellular inflammatory pathway (15).

In our study, there was no significant difference in the renal IL-10 mRNA expression after HS compared to the sham group. EPO was able to increase IL-10 mRNA expression in the kidney tissue compared to the sham and HS groups. Several studies reported that IL-10 exerts improving effects in the outcome of ischemia-reperfusion type injuries (16,17). It seems that after EPO administration, endogenous IL-10 production is involved in a normal anti-inflammatory response that reduces the damaging effects of the pro-inflammatory cascade. IL-10 attenuates inflammation by suppressing the neutrophil and monocyte activation, reduction in the activation of NF-κB, which all are involved in the kidney damage (18). It is also believed that IL-10 inhibits cytokine synthesis such as IL-6 (18).

## 6. Conclusions

In conclusion, we found that pre-treatment with EPO attenuates renal injury in rats subjected to HS. EPO exerts its improving effects on the kidney tissues, in part, due to the inflammatory gene modifications including reduction of pro-inflammatory cytokines TNF-α and IL-6 and increase in anti-inflammatory IL-10 mRNA expression.

## Authors’ contribution

All authors contributed to the design of the research. MR conducted the experiments, analyzed the data and prepared the primary draft. MK and BS edited the manuscript.

## Conflicts of interest

The authors declared no competing interests.

## Funding/Support

This work was supported by a grant from Tehran University of Medical Sciences (Grant #25840).
